# Progressive Cognitive Impairment Evolving to Dementia Parallels Parieto-Occipital and Temporal Enlargement in Idiopathic Chronic Hydrocephalus: A Retrospective Cohort Study

**DOI:** 10.3389/fneur.2015.00015

**Published:** 2015-02-24

**Authors:** Paolo Missori, Antonio Currà

**Affiliations:** ^1^Department of Neurology and Psychiatry, Neurosurgery, Policlinico Umberto I, “Sapienza” University of Rome, Rome, Italy; ^2^Department of Medico-Surgical Sciences and Biotechnologies, Neurology, A. Fiorini Hospital, “Sapienza” University of Rome, Polo Pontino, Terracina, Italy

**Keywords:** dementia, hydrocephalus, hydrodynamics, idiopathic, ventricle

## Abstract

Little is known regarding progressive enlargement of the ventricular system in symptomatic patients or asymptomatic subjects. Before eventual surgical treatment, we evaluated the clinical and radiological features of an extremely rare group of patients with idiopathic chronic hydrocephalus (ICH) and cognitive impairment evolving to dementia (*n* = 11), and an extremely rare group of asymptomatic or minimally symptomatic adults (AMSA) with ventricular enlargement (*n* = 10). We quantified changes over time in the ventricular frontal, occipital, and temporal horns by measuring the Evans’ index plus a parieto-occipital ratio and a temporal ratio, and their percentage of progression. Cerebral ventricles expanded over very long term in both demented patients with ICH and in AMSA. In AMSA, frontal enlargement predominated, whereas demented patients showed predominant parieto-occipital (*p* = 0.00) and temporal (*p* = 0.00) enlargement that progressed faster than in AMSA (*p* = 0.00). In ICH, progression of cognitive impairment parallels ventricular parieto-occipital and temporal horn enlargement. Limitations of this study are the retrospective nature, the non-uniform use of neuropsychological tests, the reduced sample size due to the extremely stringent enrollment criteria, the inability to determine the precise rate of progression.

## Introduction

Progressive enlargement of the ventricular system during aging is a common finding in the brain parenchyma. The ventricular system also enlarges progressively in a number of neurodegenerative diseases, such as Alzheimer’s disease (AD), Parkinson’s disease, and progressive supranuclear palsy, as well as in vascular dementia (VD), but specifically in idiopathic chronic hydrocephalus (ICH). Despite specific neurological presentation, all of these conditions share some clinical signs, such as cognitive impairment, gait disturbance, and urinary sphincter abnormalities. In ICH, in which the cerebral ventricles enlarge while maintaining normal cerebrospinal fluid (CSF) pressure (so called normal pressure hydrocephalus) – the coexistence of the three signs characterizes the neurological picture (i.e., the Hakim’s triad) (1).

Certainly, among the clinical signs that accompany ventricular enlargement in ICH, the most worrisome is cognitive impairment, that in untreated patients progresses and can evolve into dementia. For this reason, ICH is considered a form of treatable dementia, making it critical that physicians recognize and surgeons treat this neurological condition. The clinical relevance of evolving dementia in patients with hydrocephalus is clear when one considers that ICH is common in people over 60 years old, and conservative estimates indicate that the prevalence of idiopathic normal pressure hydrocephalus is 21.9/100,000 and the incidence is 3.74/100,000 (2, 3).

Neuroimaging using computed tomography (CT) and magnetic resonance imaging (MRI) is critical for identifying ventricular enlargement. The Evan’s index (EI), which is determined by dividing the maximum width of the frontal horns by the maximum width of the inner table of the cranium at the level of the frontal horns, is the key measure for radiological diagnosis of hydrocephalus (4). International guidelines recommend that an EI value >0.3 be used for the diagnosis of ICH (5, 6).

Unfortunately, diagnosing hydrocephalus is insufficient for identifying patients who may benefit from treatment, as it is neither clear when ventricular enlargement becomes symptomatic nor how chronic hydrocephalus leads to dementia. Since early shunting is more effective than late shunting in treating the triad of symptoms (7), it would be very helpful clinically to have reliable predictors of which asymptomatic adults will develop ICH, and/or which patients with ICH will develop dementia.

## Materials and Methods

In this retrospective cohort study, over an 8-year period (2002–2010), we recruited an extremely rare group of 21 study participants based on neuroimaging evidence of present or previous (brain scan performed at least 4 years prior) ventricular enlargement, defined as EI ≥0.3. All subjects were analyzed before an eventual surgical treatment and grouped according to the presence or absence of neurological symptoms included in the Hakim’s triad. First, subjects were categorized as being cognitively impaired or cognitively intact. Cognitive impairment was defined as the presence of one or more of the following: psychomotor slowing, impaired recall of recent events, executive dysfunction (i.e., impairment in multistep procedures, working memory, or formulation of abstractions), or behavioral or personality changes. The individuals enrolled for the study as “patients” were in charge in neurological centers with a negative or inconclusive diagnosis of probable AD, fronto-temporal dementia (FTD), VD, or movement-disorder with dementia (MD-D, including Parkinson’s disease dementia, dementia with Levy bodies, progressive supranuclear palsy, cortical basal degeneration, etc.) as well vitamin B12 deficiency, thyroid dysfunction, or other neurodegenerative diseases. All patients with cognitive impairment had undergone – in the neurological centers they were in charge and that referred us the patient for ventricular enlargement – neuropsychological testing [including mini-mental state examination (MMSE), frontal assessment battery, Rey’s 15 words immediate and delayed recall – alternative forms, Wisconsin card sorting test, trail making, attentive matrices, analogies tests, digit span forward and backward tasks, etc.] in variable settings and period of diseases. Since the aim of the work was to identify and grossly quantitate cognitive impairment in patients not otherwise classified as having a dementing disease, we decided to include only scores from MMSE, performed in all subjects (8). Each cognitively impaired patient was graded as having severe (<10 points), moderate (10–19 points), or mild (20–24 points) cognitive impairment. Cognitively intact subjects had MMSE score >26. All participants were further categorized according to the presence or absence of gait disturbance or urinary sphincter abnormalities, neurological signs common to diseases that manifest with ventricular enlargement, but peculiarly coexisting in ICH. Gait disturbance or unsteady gait was diagnosed if the subject exhibited two or more of the following signs: decreased step height, decreased step length, decreased cadence/low speed of walking, increased trunk sway during walking, widened standing base, impaired walking balance, spontaneous or provoked retropulsion, freezing phenomena, altered tandem gait (>2 corrections for 8 steps). More severe gait disturbance was diagnosed when the subject could walk only with help or when he/she was wheelchair bound. Urinary sphincter abnormalities were diagnosed when urgent micturition or urinary incontinence were present (Table 1).

**Table 1:** **Clinical features of the study participants**.

ICH-CI patients							AMSA					
*T*_previous_			*T*_last_				*T*_last_			*T*_previous_		
Cognitive	Gait	Urinary	Cognitive	Gait	Urinary	Case	Cognitive	Gait	Urinary	Cognitive	Gait	Urinary
+	−	+	++	++	++	1	−	−	−	−	−	−
+	−	−	+++	+	++	2	−	−	−	−	−	−
+	−	−	+++	++	++	3	−	−	−	−	−	−
+	−	+	++	++	+	4	−	−	+	−	−	−
+	−	−	+++	+++	++	5	−	+	−	−	−	−
+	−	−	+++	+++	++	6	−	+	−	−	−	−
+	−	−	+++	++	++	7	−	−	−	−	−	−
+	−	+	+++	+++	++	8	−	−	+	−	−	−
+	−	−	++	++	++	9	−	−	−	−	−	−
+	−	−	++	++	+	10	−	+	−	−	−	−
+	−	−	++	+++	++	11						

*Symptoms at two time points, *T*_previous_ and *T*_last_, in idiopathic chronic hydrocephalus patients with cognitive impairment (ICH-CI) and asymptomatic or minimally symptomatic adults (AMSA). Cognitive impairment was graded according to mini-mental state examination MMSE score: severe (<10 points), 3 patients; moderate (10–19 points), 5 patients; or mild (20–24 points), 3 patients*.

*Gait impairment: unsteady +; walking only with help ++; bedridden +++. Urinary impairment: urgent micturition +; urinary incontinence ++. “−”means unaltered*.

When ventricular enlargement accompanied at least two neurological signs and symptoms that were related to Hakim’s triad, the subjects were diagnosed as “ICH” patients (5, 6). When ventricular enlargement was present with no symptoms or just one non-cognitive very mild symptom associated with the triad (e.g., the subject lived independently and had no restrictions in daily activities), the subjects were diagnosed as “asymptomatic or minimally symptomatic adults” (AMSA).

Exclusion criteria were as follows: a history of congenital hydrocephalus, central nervous system (CNS) hemorrhage, venous sinus thrombosis, CNS infections, and obstructing lesions. We also excluded patients with cognitive impairment plus ventricular enlargement whose clinical progression, associated neurological features, and neuroimaging data were suggestive or indicative of a dementing disorder other than ICH.

The extremely stringent clinical and radiological criteria adopted for enrollment limited the number of study participants markedly, thereby causing the sample size to be of convenience. Subjects were rare because an enlarged ventricular system often accompanies neurological signs or symptoms; patients were rare because of the paucity of cognitively impaired subjects with ventricular enlargement and negative criteria for a probable dementing disease.

**Figure 1:**
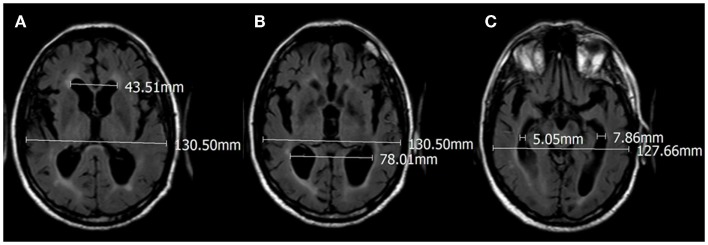
**Seventy-eight-year-old male with cognitive impairment before surgical treatment, with progressive gait disturbance and urgent urination**. **(A)** Evan’s index 43.51/130.50 = 0.33; **(B)** POR 78.01/130.50 = 0.59; **(C)** TR 5.05 + 7.86/127.66 = 0.10.

Brain scans were performed in different neuroimaging centers all using standardized techniques of image acquisition. Axial brain slices oriented along the orbitomeatal line, allowed a comparable anatomy of brain structures from different patients. Using the neuroimaging data collected at two time points *T*_last_ and *T*_previous_, we calculated the EI value by dividing the maximum width of the frontal horns by the maximum width of the inner table of the cranium at the level of the Monro’s foramens in the frontal horns (Figure 1A). We also calculated two additional measures, which we defined for the purposes of this study. The first measure was the ventricular parieto-occipital ratio (POR), which was the ratio of the maximum width of the occipital horns at the atrium to the maximum width of the inner table of the cranium at the same level (Figure 1B). The second measure was the ventricular temporal ratio (TR), which was the ratio of the maximum width of the temporal horns at the level of maximal convexity of the hippocampus and the maximum width of the inner table of the cranium at the same level (Figure 1C) (Table 2). For all three indexes, i.e., EI, POR, and TR, we also calculated the percentage of progression per year (%PR) using the formula [(Index_last_ − Index_previous_)/follow-up in months] × 12 × 100. CSF flow studies with cisternography or MRI were performed in six patients with cognitive impairment and in four subjects without symptoms. The resulting was not conclusive for any clear alteration of CSF flow dynamics and therefore excluded from the study.

**Table 2:** **Radiological features of the study participants**.

Group	Age_previous_	Age_last_	FUP	EI_previous_	EI_last_	POR_previous_	POR_last_	TR_previous_	TR_last_
ICH-CI-1	59	63	54	0.31	0.33	0.50	0.61	0.07	0.16
ICH-CI-2	68	74	69	0.32	0.35	0.53	0.57	0.09	0.21
ICH-CI-3	58	64	73	0.28	0.35	0.56	0.64	0.14	0.30
ICH-CI-4	82	88	73	0.35	0.38	0.52	0.55	0.08	0.16
ICH-CI-5	66	73	82	0.30	0.28	0.52	0.58	0.11	0.22
ICH-CI-6	71	80	106	0.36	0.36	0.62	0.67	0.11	0.17
ICH-CI-7	56	68	142	0.34	0.40	0.62	0.67	0.05	0.13
ICH-CI-8	66	70	57	0.33	0.38	0.53	0.63	0.06	0.13
ICH-CI-9	72	77	65	0.27	0.30	0.56	0.65	0.04	0.10
ICH-CI-10	65	75	116	0.28	0.32	0.48	0.58	0.04	0.15
ICH-CI-11	55	65	116	0.25	0.33	0.53	0.60	0.04	0.07
AMSA-1	77	81	148	0.38	0.40	0.61	0.64	0.12	0.16
AMSA-2	56	60	48	0.36	0.36	0.51	0.54	0.03	0.04
AMSA-3	66	71	67	0.37	0.39	0.53	0.54	0.10	0.15
AMSA-4	57	63	79	0.45	0.44	0.64	0.64	0.09	0.09
AMSA-5	60	72	144	0.35	0.38	0.61	0.68	0.13	0.22
AMSA-6	61	74	154	0.28	0.36	0.44	0.50	0.08	0.11
AMSA-7	51	66	176	0.37	0.39	0.56	0.57	0.16	0.13
AMSA-8	42	75	385	0.30	0.37	0.53	0.59	0.03	0.06
AMSA-9	30	40	120	0.27	0.31	0.50	0.54	0.02	0.05
AMSA-10	55	61	65	0.31	0.33	0.50	0.59	0.03	0.04

*Age (years); FUP (follow-up between time points *T*_previous_ and *T*_last_, months); EI (Evan’s index, obtained by dividing the maximum width of the frontal horns by the maximum width of the inner table of the cranium at the level of the frontal horns); POR (parieto-occipital ratio, obtained by dividing the maximum width of the occipital horns at the atrium by the maximum width of the inner table of the cranium at the same level); TR (temporal ratio, obtained by dividing the maximum width of both temporal horns at the level of maximal convexity of the hippocampus by the maximum width of the inner table of the cranium at the same level). (ICH-CI) patients with idiopathic chronic hydrocephalus and dementia; (AMSA) asymptomatic or minimally symptomatic adults*.

We used the statistical package, version 10 for Windows (StatSoft Inc., OK, USA), for all analyses. We subjected variables to separate analysis of variance (ANOVA) analyses with between factor “group” (patients and asymptomatic subjects) and repeated measure factor “time” (last and previous). The %PR values were subjected to between-group multivariate ANOVA with the factor “group.” The Tukey honestly significant difference test was used for *post hoc* analyses. *p* Values <0.01 were considered significant.

## Results

All cognitively impaired patients also had gait and urinary disturbances, therefore they were labeled as “ICH-CI” (i.e., ICH-cognitively impaired; *n* = 11). After measuring CSF open pressure, all of them fitted the criteria for diagnosis of probable iNPH. According to the MMSE scores three ICH-CI patients had severe cognitive impairment (MMSE <10 points), five patients had moderate cognitive impairment (MMSE 10–19 points), and three patients had mild cognitive impairment (MMSE 20–24 points).

Cognitively intact subjects (MMSE score >26, *n* = 10) were asymptomatic or minimally symptomatic (5 out 10 had either very mild gait disturbance or urinary sphincter abnormalities), and all lived independently and had no restrictions in their daily activities.

The time elapsed between scans performed at the two time points *T*_last_ and *T*_previous_ ranged from 48 to 385 months (average time period 111.4 month). Subjects were examined clinically at *T*_last_, whereas the clinical status at *T*_previous_ was recorded based on recollections of the subjects and their relatives (Table 1).

### ICH-CI patients

In patients with ICH-CI (mean age at *T*_last_, 72.4 ± 7.5 years; mean age at *T*_previous_, 65.3 ± 8.6 years; 6 women), the clinical features at *T*_last_ are shown in Table 1. At *T*_previous_, the first symptoms were subtle changes in mental state, such as loss of short-term memory, slowed responses, decreased reactions, and new-onset anxiety. These symptoms were all reported as unexpected behavioral changes by relatives. From *T*_previous_ to *T*_last_, the patients generally showed deteriorating neurological conditions, with variable declines in cognitive functions, gait disturbance, and sphincter abnormalities.

At *T*_last_, the mean EI in the ICH-CI group (CTs, *n* = 4; MRI, *n* = 7) was 0.34 ± 0.036 (range 0.28–0.40), the mean POR was 0.61 ± 0.04 (range 0.55–0.67), and the mean TR was 0.16 ± 0.06 (range 0.07–0.30) (Table 2). At *T*_previous_ (mean 86.64 ± 28.77 months follow-down, range 54–142 months), the mean EI in the ICH-CI group was 0.31 ± 0.03 (range 0.25–0.36), the mean POR was 0.54 ± 0.04 (range 0.48–0.62), and the mean TR was 0.07 ± 0.03 (range 0.04–0.14).

### Asymptomatic or minimally symptomatic adults

In AMSA with incidental findings of ventricular enlargement on brain imaging studies at *T*_last_ (mean age at *T*_last_, 66.30 ± 11.4 years; mean age at *T*_previous_, 55.50 ± 12.8 years; 2 women), the mean EI (CTs, *n* = 5; MRI, *n* = 5) was 0.37 ± 0.03 (range 0.31–0.44), the mean POR was 0.58 ± 0.06 (range 0.50–0.68), and the mean TR was 0.10 ± 0.06 (range 0.04–0.22). At *T*_previous_ (mean 138.60 ± 97.24 months follow-down, range 48–385 months), the mean EI was 0.34 ± 0.05 (range 0.27–0.45), the mean POR was 0.54 ± 0.06 (range 0.44–0.64), and the mean TR was 0.08 ± 0.05 (range 0.02–0.16) (Table 2).

### Comparison between groups from *T*_previous_ to *T*_last_

**Figure 2:**
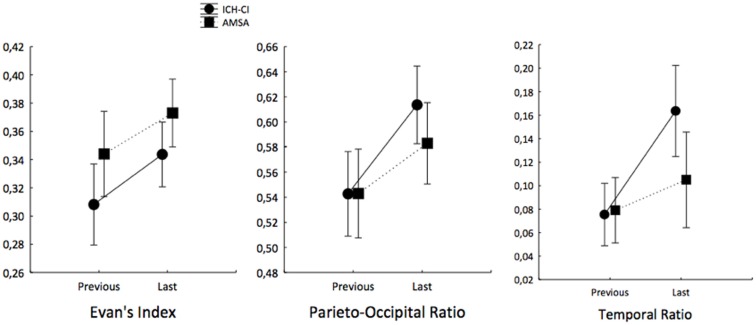
**Time course of the radiological indexes of ventricular dilatation in ICH-CI patients before surgical treatment and AMSA from *T*_previous_ to *T*_last_**. Note that all indexes increased from *T*_previous_ to *T*_last_ in both groups, but only POR and TR increased more in ICH-CI patients than in AMSA. Moreover, EI was lower in ICH-CI patients than in AMSA.

Analysis of variance testing of the EI values showed a main effect for the factor time [*F*_(1, 19)_ = 26.23, *p* = 0.00]. *Post hoc* analysis showed that the EI increased from *T*_previous_ to *T*_last_ in both AMSA and ICH-CI patients (Figure 2, left panel). There was a marginally significant group effect [*F*_(1, 19)_ = 3.7, *p* = 0.07], indicating that AMSA had higher EI values than ICH-CI patients.

Analysis of variance testing of the POR values showed a main effect for the factor time [*F*_(1, 19)_ = 81.12, *p* = 0.00], and a significant interaction of group with time [*F*_(1, 19)_ = 6.20, *p* = 0.02]. *Post hoc* analysis showed that the POR increased from *T*_previous_ to *T*_last_ in both groups, but that the POR increased more in ICH-CI patients than in AMSA (Figure 2, center panel).

Analysis of variance testing of the TR values showed a main effect for the factor time [*F*_(1, 19)_ = 59.51, *p* = 0.00] and a significant interaction of group with time [*F*_(1, 19)_ = 17.65, *p* = 0.00]. *Post hoc* analysis showed that the TR increased from *T*_previous_ to *T*_last_ in both groups, but that the TR increased more in ICH-CI patients than in AMSA (Figure 2, right panel).

**Figure 3:**
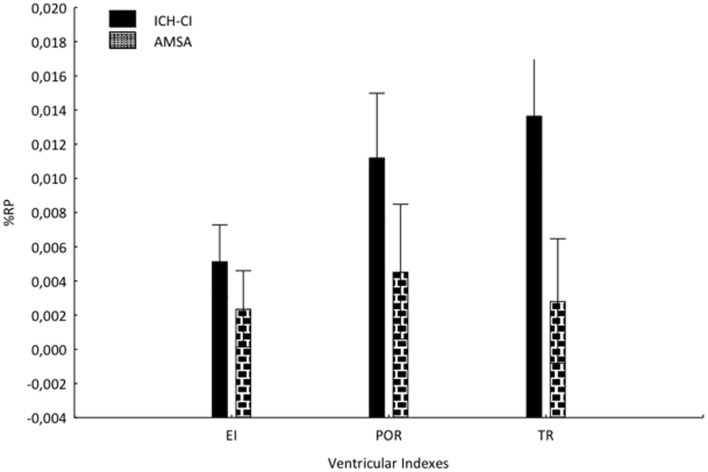
**The percentage rate of progression (RP) per year for each ventricular index in both groups**. The RP was calculated using the formula: [(Index_last_ − Index_previous_)/follow-up in months] × 12 × 100.

Analysis of variance testing of the %P for each index showed a main effect for the factor group [Wilks λ = 0.46578, *F*_(3, 17)_ = 6.4992, *p* = 0.00396]. *Post hoc* analysis showed that POR (*p* = 0.01) and TR (*p* = 0.0002), but not EI (*p* = 0.07), progressed faster in ICH-CI patients than in AMSA (Figure 3).

## Discussion

In this clinical retrospective radiological observational study, we found that the size of the cerebral ventricles increased over very long term in both ICH-CI patients and AMSA. How much and how quickly the cerebral ventricles enlarged differed in the two groups, i.e. in individuals with neurological symptoms compared to those without neurological symptoms. In AMSA, the frontal ventricles enlarged the most, whereas patients with ICH-CI showed predominant parieto-occipital and temporal enlargement. The rate of ventricular enlargement differed both between groups (ICH-CI vs. AMSA) and in different parts of the ventricular system. Parieto-occipital and temporal enlargement progressed faster in ICH-CI patients than in AMSA, whereas frontal enlargement progression was similar in the two groups. Ventricular changes can be followed easily by measuring two new simple – everywhere applicable – radiological indexes, POR and TR, which were more reliable than EI for identifying subjects with hydrocephalus that developed dementia.

Here, we report that ventricular enlargement differs in different brain regions and is associated with distinct clinical patterns. In the ICH-CI patients before an eventual surgical treatment, neurological decline (i.e., motor, sphinterial, and cognitive) paralleled ventricular system enlargement over time, although the mechanisms underlying this association remain unclear. CSF dynamics may be an important factor in this phenomenon.

In the human brain, Pascal’s principle predicts that the ventricular size will remain unchanged throughout life, explaining why the CSF pressure is uniform in the ventricular system and subarachnoid spaces (there is no flow, only diffusion) (9, 10). Physiological or pathological conditions that alter CSF absorption may result in slight changes in the mean CSF intra-ventricular volume and pressure. Due to the variable anatomical structure of the ventricular system, which is organized as sequential cavities, pressure mini-gradients can be generated inside the ventricular and extra-ventricular cavities. These mini-gradients result in CSF flow (11, 12), leading to the well known MRI finding of CSF flow-void in which the CSF pressure is minimal (13, 14).

If a pathogen triggers an ongoing physiological reaction, then the ventricular size and extra-ventricular cavities such as the Sylvian cisterns may minimally, but progressively, increase, thereby producing both internal and “external hydrocephalus” (15, 16).

In our ICH-CI patients, progressive cognitive impairment paralleled parieto-occipital and temporal dilatation. This observation is in accordance with cerebral blood flow studies in ICH patients showing that hypoperfusion in the parieto-occipital brain areas correlates with dementia severity (17–19). Because the pathology underlying dementia mainly involves the neural structures in the parietal–temporal–occipital junction, the topographical relationship between expanding ventricles and dysfunction in adjacent regions of the brain raises the question of whether ventricular enlargement results from hydrocephalus or *ex vacuo* changes. Although this may represent the chicken-egg dilemma of neurological practice (3, 20), we propose that at least in some of ICH patients who develop dementia, the topographical relationship is causative, possibly dependent on the CSF tension that is exerted on the ventricle walls. In such ICH-CI patients, the ventricle mini-gradients and flows – as well as changes in the clearance mechanisms operated by CSF circulation – represent the *primum movens*, rather than the consequences, of neural regressive abnormalities that ultimately lead to dementia (18). This is the main difference compared to the ventriculomegaly that frequently accompanies neurodegenerative diseases and normal aging. At least in some ICH-CI patients, internal hydrocephalus therefore precedes and is greater than external hydrocephalus, whereas in neurodegenerative dementing disorders ventricular dilatation and cortical subarachnoid spaces enlargement are concomitant. We speculate in these patients that the disease spectrum, which ranges from asymptomatic ventriculomegaly to dementia with chronic hydrocephalus, is the result of an evolving hydrodynamic CSF disease. In this condition, intervening factors modify the age-related changes in CSF dynamics, thereby altering the function of neural structures contiguous with the intra- and extra-ventricular space, possibly by inducing a reversible neuronal injury that has the potential for recovery when pathologic CSF circulation is corrected (21).

In this study, we found that POR and TR were proportional to cognitive impairment and more useful measures than EI for identifying ICH patients with progressive cognitive impairment evolving to dementia. William Evans introduced the EI ratio to determine the normal limits of ventricular size and to differentiate normal from hydrocephalic patients (4). When CT replaced pneumoencephalography for diagnosing hydrocephalus, EI reliability was demonstrated for both of these two neuroimaging techniques (22, 23). Currently, EI is considered the gold standard for monitoring the degree and the progression of frontal ventricular dilatation in adults with ICH (5, 6).

Since the study design was retrospective, we could not choose the time elapsing between consecutive neuroimaging studies and not assess the progression pattern, but not progression itself, that was the very aim of the study. In our series, no demented patient at *T*_last_ showed a POR < 0.55 and a TR < 0.07. Therefore, we propose these values as the cut-off values for identifying subjects who should be strictly monitored, regardless of whether they are symptomatic. By monitoring changes in EI, POR, and TR over time, patients with evolving neurological symptoms can avoid supplemental invasive preoperative prognostic tests and be considered for surgical intervention. After surgery, 80% of patients improve clinically, although remarkably, the size of their ventricular system remains unchanged as measured by EI (24, 25). Since the ventricular system in ICH-CI patients enlarged in a non-uniform manner, after eventual surgical treatment repeatedly measuring POR and TR in addition to EI may help determine how the shunt works and allow decision-making.

Finally, we acknowledge some limitations of this study, i.e., its retrospective nature, the non-uniform use of neuropsychological tests, the reduced sample size due to the extremely stringent enrollment criteria, the inability to determine the precise rate of progression.

## Conclusion

Cerebral ventricles expand over very long term in both ICH and asymptomatic ventriculomegaly. In patients with ICH who develop dementia, the ventricular system enlarges progressively but non-uniformly, leading to disproportionate parieto-occipital and temporal dilatation. Ventricular expansion is best monitored by measuring the EI, POR, and TR. All asymptomatic adults with ventriculomegaly having POR > 0.55 and TR > 0.07 should be clinically and radiologically followed up to prevent delayed diagnosis and treatment.

## Conflict of Interest Statement

The authors declare that the research was conducted in the absence of any commercial or financial relationships that could be construed as a potential conflict of interest.
